# Expert Evaluation of the Perceived Accuracy, Relevance, and Safety of Large Language Model–Generated Patient Information in Geriatrics: Cross-Condition Study

**DOI:** 10.2196/91369

**Published:** 2026-05-04

**Authors:** Sebastian Martini, Sabine Schluessel, Ughur Aghamaliyev, Michaela Rippl, Linda Deissler, Olivia Tausendfreund, Desiree Nuebler, Katharina Mueller, Ralf Schmidmaier, Michael Drey

**Affiliations:** 1 Department of Medicine IV, Geriatrics LMU Munich Hospital Ludwig-Maximilians-Universität München Munich, Bavaria Germany; 2 Department of General, Visceral, and Transplant Surgery LMU Munich Hospital Ludwig-Maximilians-Universität München Munich, Bavaria Germany

**Keywords:** large language models, LLMs, ChatGPT, geriatric medicine, patient education, medical informatics, expert consensus, artificial intelligence in health care

## Abstract

**Background:**

Large language models (LLMs) are increasingly used to generate patient-oriented medical information. In geriatrics, such information must balance accuracy, relevance, and safety, as older adults may be particularly susceptible to misleading or harmful advice. However, systematic evaluations of expert perceptions across multiple geriatric conditions remain limited.

**Objective:**

This study aimed to explore geriatricians’ perceptions of the accuracy, relevance, and potential harm of LLM-generated patient information across common geriatric conditions and to examine variability and interrater agreement in expert ratings.

**Methods:**

In this cross-sectional expert rating study, 10 geriatricians evaluated 50 LLM-generated statements covering 5 geriatric conditions (sarcopenia, osteoporosis, urinary incontinence, depression, and dementia). Statements addressed diagnostic, etiological, prognostic, risk-related, and therapeutic aspects. Experts rated perceived accuracy, relevance, and potential harm using 5-point Likert scales. Rating distributions were summarized using medians and IQRs. The Kendall coefficient of concordance (W) was used exploratorily to assess agreement in the relative ordering of statements within predefined strata. Readability was assessed using Flesch-Kincaid Grade Level and Flesch Reading Ease.

**Results:**

Expert ratings indicated high perceived accuracy (median 4.32, IQR 4.01-4.59 and perceived relevance (median 4.51, IQR 4.06-4.66), while perceived potential harm remained low (median 1.59, IQR 1.17-1.92). IQR values ranged from 0.00 to 1.38 with most values clustering below 0.5, indicating limited dispersion in expert ratings. Agreement in the relative ordering of statements varied across domains, with W values ranging from 0.27 to 0.62 (median 0.53, IQR 0.46-0.58), indicating moderate concordance. No statements combined low perceived accuracy with high perceived potential harm. Readability analysis indicated generally accessible language, with a median Flesch-Kincaid Grade Level of 8.3 (IQR 7.4-9.6) and a median Flesch Reading Ease score of 60.8 (IQR 50.1-66.9).

**Conclusions:**

LLM-generated patient information for common geriatric conditions was rated as largely accurate and relevant, with low potential harm in typical scenarios. Variability in expert emphasis and the exploratory nature of agreement analyses highlight the limitations of perception-based evaluation. Future studies should incorporate guideline-based validation, readability optimization, and patient-centered outcomes to more comprehensively evaluate the safety and suitability of LLM-generated information for geriatric patient education.

## Introduction

Geriatric patients represent a rapidly growing population affected by prevalent conditions such as sarcopenia, osteoporosis, urinary incontinence, depression, and dementia [1]. Patients and caregivers frequently seek health information online to better understand diagnostic procedures, disease mechanisms, therapeutic options, associated risks, and prognosis [2]. However, available information is often fragmented, inconsistent, or insufficiently tailored to varying levels of medical knowledge [3]. As a result, the complexity and framing of medical content may not align with the informational needs of older adults, particularly in the context of multimorbidity.

In this setting, large language models (LLMs) such as ChatGPT (OpenAI) are increasingly used to generate health-related information for patients and caregivers [4,5]. Unlike static online resources, these systems synthesize responses to user queries and may adapt explanations to the context of the question [6]. Their ability to produce fluent and coherent text has raised interest in their potential role in patient education and decision support. At the same time, concerns persist regarding the accuracy, relevance, and safety of LLM-generated medical content, particularly when responses appear plausible but contain subtle inaccuracies or omit clinically important caveats [7].

These concerns are especially relevant in geriatrics. Older adults often manage multiple chronic conditions, polypharmacy, and functional impairments, increasing susceptibility to misleading or incomplete medical advice. Even minor inaccuracies or ambiguous recommendations may result in delayed care seeking, inappropriate self-management, or medication-related harm [8]. Consequently, evaluating LLM-generated patient information in geriatric contexts requires careful consideration not only of perceived accuracy but also of perceived relevance and potential harm.

Previous studies assessing LLM performance in medicine have predominantly focused on factual accuracy using benchmark questions, examinations, or guideline-based comparisons [9-11]. While valuable, these approaches may not fully capture how clinicians perceive the usability and safety of patient-oriented information in real-world contexts. Expert rating studies provide a complementary perspective by capturing clinician judgments of perceived accuracy, relevance, and risk, particularly where formal gold standards are difficult to operationalize. Despite the high prevalence of multimorbidity in older adults, most evaluations of artificial intelligence (AI)–generated patient information have focused on single diseases rather than cross-condition assessment [12,13]. Likert-scale ratings summarize absolute judgments but provide limited insight into agreement among raters. Measures of dispersion describe variability in ratings, whereas concordance statistics capture consistency in relative ordering. These complementary approaches provide a more comprehensive understanding of expert evaluation.

Against this background, this study provides an exploratory expert-based evaluation of LLM-generated patient information across 5 common geriatric conditions: sarcopenia, osteoporosis, urinary incontinence, depression, and dementia. Geriatricians rated responses addressing diagnostic, etiological, prognostic, risk-related, and therapeutic aspects with respect to perceived accuracy, perceived relevance, and perceived potential harm. The aim was not to establish objective clinical correctness or clinical prioritization but to examine patterns of perceived quality, variability, and agreement in expert judgments across conditions and content domains.

## Methods

### Study Design and Workflow

**Figure 1:**
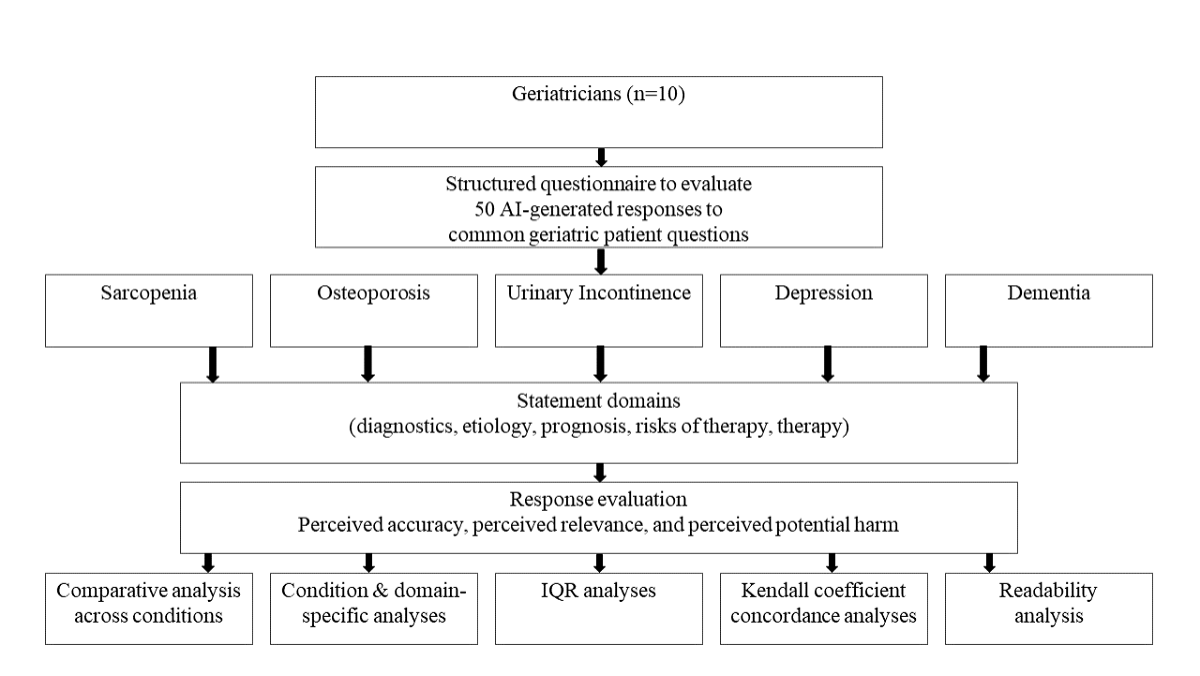
Study workflow and survey design. Geriatricians evaluated 50 large language model–generated responses to common geriatric patient questions across 5 geriatric conditions (sarcopenia, osteoporosis, urinary incontinence, depression, and dementia). For each condition, statements addressed diagnostics, etiology, prognosis, risks of therapy, and therapy. Responses were rated with respect to perceived accuracy, perceived relevance, and perceived potential harm. AI: artificial intelligence.

This study was designed as a cross-sectional expert evaluation of LLM-generated responses to common geriatric patient questions. The overall study workflow is summarized in Figure 1. Briefly, frequently asked geriatric questions were identified by a panel of geriatricians; submitted to ChatGPT using a standardized procedure; and subsequently evaluated by expert reviewers with respect to perceived accuracy, perceived relevance, and perceived potential harm. Readability of generated responses was assessed using established metrics (Flesch Reading Ease and Flesch-Kincaid Grade Level) to provide complementary information on accessibility of the content. In addition, demographic and professional background information of participating experts was collected. The study represents an exploratory assessment of expert perceptions and did not aim to establish objective clinical correctness or guideline adherence.

### Identification and Selection of Geriatric Patient Questions

A panel of 6 board-certified geriatricians identified frequently asked questions encountered in routine geriatric practice through structured group discussion. Questions covered 5 common geriatric disease domains: sarcopenia, osteoporosis, urinary incontinence, depression, and dementia. The aspects covered were diagnostics, etiology, prognosis, risks of therapy, and therapy.

The identified questions were subsequently evaluated by each panel member using a predetermined point-based system that reflected their perceived frequency in routine clinical practice. For each question, geriatricians assigned a score of 1 (infrequently encountered), 2 (moderately frequent), or 3 (very frequently encountered). Scores were aggregated across all panel members, and questions with the highest total scores were selected for inclusion. The final sample size of 50 questions was predetermined to ensure representation across diseases. These 50 questions were retained for the subsequent generation and evaluation of LLM responses. This approach ensured that the evaluated questions reflected common information needs among geriatric patients and caregivers. Consequently, the resulting distribution across content domains was not uniform, reflecting the pragmatic selection based on clinical relevance and frequency.

### Generation of LLM Responses

All selected questions were submitted to ChatGPT (version 5.1; OpenAI) between November 13 and 18, 2025. A paid subscription tier (ChatGPT Plus) was used. For each question, a new and independent chat session was initiated. As the standard web interface does not allow manual adjustment of generation parameters, default model settings (eg, temperature and sampling parameters) were applied. Questions were submitted using a standardized input procedure consisting solely of the respective patient question listed in Multimedia Appendix 1. No additional instructions, role assignments, or system prompts were included in the input field.

However, within the ChatGPT personalization settings, a background description was specified indicating that the user was aged >70 years and had several health problems (“I am over 70 years old and have several health issues.”). This contextual setting was used to approximate a realistic interaction scenario in which older adults seek medical information from conversational AI systems.

Each LLM-generated response consisted of a short paragraph addressing a patient question. For the purpose of expert evaluation, each generated answer was treated as one evaluable statement. No further segmentation of responses into substatements was performed. No follow-up questions or clarifications were provided. No postprocessing or manual editing of responses was performed prior to expert evaluation. The resulting responses constituted the material evaluated in this study (Multimedia Appendix 1).

### Expert Panel and Rating Procedure

A total of 10 geriatricians with clinical experience in the care of geriatric patients participated as expert reviewers. The geriatricians came from 4 different geriatric divisions across Germany. Their demographic and professional background data, years of clinical experience, and areas of specialization were collected using a standardized questionnaire. These characteristics are summarized in Multimedia Appendix 1.

Each expert evaluated all LLM-generated responses independently with respect to 3 dimensions: perceived accuracy, perceived relevance, and perceived potential harm. Perceived accuracy was defined as the extent to which a statement appeared correct and clinically plausible based on the rater’s expertise. Relevance captured the perceived usefulness of the statement for patient education. Potential harm was defined as the likelihood of patient harm if the information were followed, including unsafe self-management, delayed care seeking, medication-related risks, or omission of clinically relevant warnings. Likert-scale responses were numerically coded for analysis only. Ratings reflected individual clinical judgment and were not intended to represent consensus-based guideline validation.

Statements were evaluated using a 5-point Likert scale with verbally anchored response options. The scale comprised the categories “strongly disagree,” “disagree,” “neutral,” “agree,” and “strongly agree,” for which numerical labels were not displayed during the rating process. Consequently, reviewers were not exposed to numeric values during the assessment process. For the purpose of statistical analysis, the responses were subsequently encoded numerically on a scale ranging from 1 (strongly disagree) to 5 (strongly agree). This encoding step was performed exclusively during data analysis and did not influence the evaluation procedure itself.

For each of the 50 questions, responses were evaluated along 3 distinct dimensions: perceived accuracy, perceived relevance, and perceived potential harm. The total number of evaluations completed by each expert amounted to 150, corresponding to 50 questions assessed across 3 distinct dimensions. To ensure data quality, given the high number of ratings, an instructed-response attention check item was included in the questionnaire as a separate multiple-choice question within the survey platform instructing participants to select “orange juice.” All reviewers responded correctly, indicating full task engagement.

### Statistical Analysis

All analyses were conducted at the level of independent expert raters to avoid item-level pseudoreplication. Each evaluated response was uniquely assigned to one disease category and one content domain; thus, every statement simultaneously belonged to a specific disease and a specific content domain.

For inferential comparisons across diseases and content domains, ratings were aggregated within each rater by calculating the mean Likert score across all responses belonging to the respective disease (or content domain) and rating dimension. Consequently, for each rating dimension, every rater contributed one aggregated value per disease and one aggregated value per content domain. Disease-specific analyses therefore reflect aggregation across content domains within each disease, whereas content-domain analyses reflect aggregation across diseases within each domain. These aggregated subject-level values served as the basis for statistical comparisons.

Given the ordinal nature of Likert-scale data and the small number of raters, nonparametric methods were used throughout. For the Friedman test, mean scores were used for within-rater aggregation across items, representing a common approach in nonparametric repeated-measures analyses to enable ranking procedures. For each item, ratings from multiple experts were first averaged to obtain a single aggregated score per item. These aggregated scores were then used to calculate medians and IQRs across items. Differences across diseases and across content domains were explored using Friedman tests with the rater as the blocking factor.

Where global tests suggested differences, pairwise Wilcoxon signed-rank tests with Bonferroni correction were applied. All tests were 2-sided, and a *P* value <.05 was considered statistically significant. Given the exploratory design and limited number of raters, inferential statistics were interpreted cautiously and used to characterize patterns rather than to infer equivalence or definitive differences.

For descriptive analyses at the statement level, ratings were summarized using medians and IQRs. These measures were used to characterize central tendency and dispersion of expert judgments across statements, diseases, and content domains. IQR values were reported descriptively to reflect the spread of expert ratings.

To explore agreement among experts regarding the relative ordering of responses within specific strata, the Kendall coefficient of concordance (W) was calculated using the *irr* package in R (version 4.5.2; R Foundation for Statistical Computing). This implementation applies a tie-corrected formulation of Kendall W, which accounts for the large number of tied ranks inherent in Likert-scale data. Analyses were performed separately within predefined strata defined by the disease–content domain–rating dimension. Within each stratum, Likert-scale ratings were converted into ordinal ranks within each rater to derive the relative ordering of responses. Ties were handled using average ranks. Agreement analyses were restricted to strata containing at least 3 responses, acknowledging that concordance estimates based on very small item counts may be unstable. Kendall W values were interpreted descriptively and considered exploratory.

Readability analyses were conducted on the full response texts prior to evaluation. Flesch Reading Ease and Flesch-Kincaid Grade Level scores were calculated using the *quanteda* package in R for each response and summarized descriptively using medians and IQRs.

### Statistical Software

All analyses were performed using R within the RStudio integrated development environment (version 2025.09.2; Posit Software). Statistical analyses were conducted to explore differences in expert ratings across diseases and content domains.

### Ethical Considerations

The ethics committee of the Ludwig-Maximilian-University Medical Faculty confirmed that formal ethics approval was not required for this study (project 25-0855 KB; October 1, 2025), as it did not involve patients or patient data. Participation of expert reviewers was voluntary, anonymous, did not include any compensation, and was obtained after informed consent. No personal identifying information was collected.

## Results

### Expert Ratings of Perceived Accuracy, Perceived Relevance, and Perceived Potential Harm

**Figure 2:**
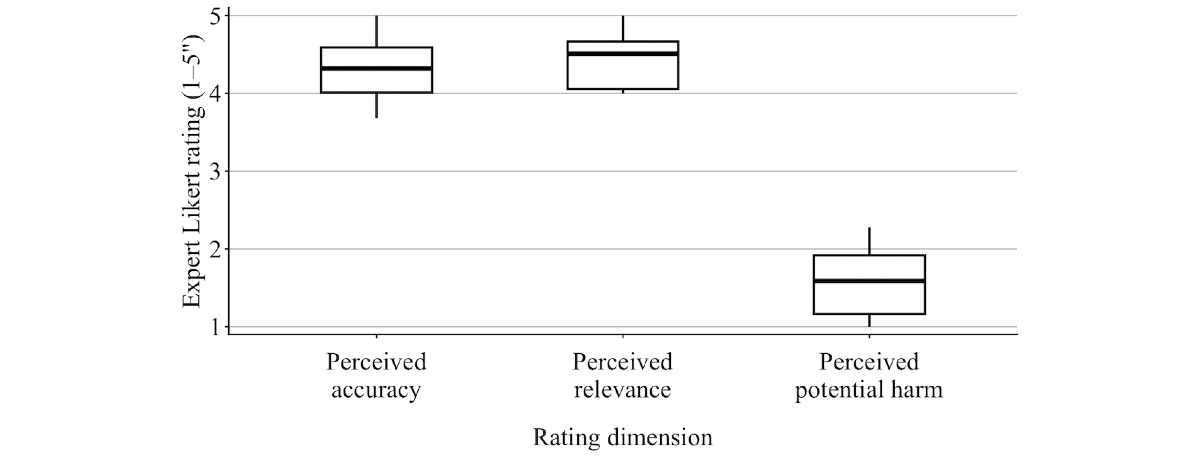
Distribution of expert ratings for perceived accuracy, perceived relevance, and perceived potential harm across all statements. Boxplots summarize statement-level ratings aggregated across raters, displaying the median and IQR; whiskers represent 1.5×IQR. Ratings are based on 5-point Likert scales (1=strongly disagree; 5=strongly agree).

Across 50 statements rated by 10 geriatricians, LLM-generated statements were rated as highly accurate (median 4.32, IQR 4.01-4.59) and relevant (median 4.51, IQR 4.06-4.66), with low perceived potential harm (median 1.59, IQR 1.17-1.92; Figure 2).

When stratified by disease, expert ratings demonstrated consistently high perceived accuracy and perceived relevance across sarcopenia, osteoporosis, urinary incontinence, depression, and dementia, accompanied by low perceived potential harm (Table 1). Median ratings for perceived accuracy and perceived relevance were uniformly located in the upper range of the Likert scale across all conditions, with overlapping IQRs. Perceived potential harm ratings remained low for all diseases and showed limited dispersion, indicating broadly consistent expert perceptions of response quality and safety across disease domains.

**Table 1:** Expert ratings of perceived accuracy, perceived relevance, and perceived potential harm stratified by disease (sarcopenia, osteoporosis, urinary incontinence, depression, and dementia)a.

Disease	Perceived accuracy, median (IQR)	Perceived relevance, median (IQR)	Perceived potential harm, median (IQR)
Sarcopenia	4.25 (3.94-4.82)	4.57 (4.16-4.76)	1.10 (1.05-1.95)
Osteoporosis	4.35 (4.12-4.54)	4.42 (4.23-4.59)	1.53 (1.12-1.98)
Urinary incontinence	4.43 (4.07-4.47)	4.60 (4.18-4.84)	1.70 (1.02-2.00)
Depression	4.28 (4.03-4.58)	4.23 (4.05-4.71)	1.67 (1.08-2.00)
Dementia	4.20 (3.92-4.65)	4.45 (4.05-4.77)	1.35 (1.12-2.00)

^a^Ratings are based on 5-point Likert scales and are summarized as median and IQR, reflecting the ordinal nature of the data. Values represent aggregated statement-level scores derived from expert ratings.

When stratified by content domain, expert ratings showed consistently high perceived accuracy and perceived relevance across diagnostics, etiology, prognosis, risks of therapy, and therapy, accompanied by low perceived potential harm (Table 2). Median ratings for perceived accuracy and perceived relevance were generally highest for etiologic and prognostic statements, while perceived potential harm remained low across all domains, with relatively limited dispersion as reflected by the IQRs.

**Table 2:** Expert ratings of perceived accuracy, perceived relevance, and perceived potential harm stratified by content domain (diagnostics, etiology, prognosis, risks of therapy, and therapy)a.

Content domain	Perceived accuracy, median (IQR)	Perceived relevance, median (IQR)	Perceived potential harm, median (IQR)
Diagnostics	4.15 (3.70-4.47)	4.55 (4.03-4.80)	1.60 (1.15-1.87)
Etiology	4.48 (4.00-4.70)	4.40 (4.03-4.72)	1.42 (1.06-1.85)
Prognosis	4.60 (4.28-4.70)	4.70 (4.55-4.97)	1.45 (1.10-1.75)
Risks of therapy	4.23 (3.90-4.52)	4.32 (4.01-4.64)	1.55 (1.24-2.08)
Therapy	4.31 (4.10-4.54)	4.32 (4.18-4.72)	1.54 (1.19-1.91)

^a^Ratings are based on 5-point Likert scales and are summarized as median and IQR, reflecting the ordinal nature of the data. Values represent statement-level scores aggregated across expert ratings.

Importantly, no statement received a combination of low perceived accuracy (median <3) and high perceived potential harm (median >3), indicating that statements judged as less accurate were not simultaneously perceived as harmful.

### Exploratory Group Comparisons

Exploratory comparisons across diseases and content domains were conducted using Friedman tests, followed by pairwise Wilcoxon signed-rank tests with Bonferroni correction where applicable.

No statistically significant differences were observed between diseases for perceived accuracy, perceived relevance, or perceived potential harm (all adjusted *P* values >.99).

The global Friedman test indicated differences across content domains for perceived accuracy (*χ*²_4_=10.2; *P*=.03) and perceived relevance (*χ*²_4_=11.5; *P*=.02). However, these differences did not remain statistically significant after Bonferroni correction in pairwise comparisons. Given the limited number of expert raters, these analyses should be interpreted cautiously. The absence of statistically significant post hoc differences does not imply equivalence but reflects limited statistical power under multiple-testing correction.

### Uncertainty of Expert Ratings (IQR-Based Analysis)

**Figure 3:**
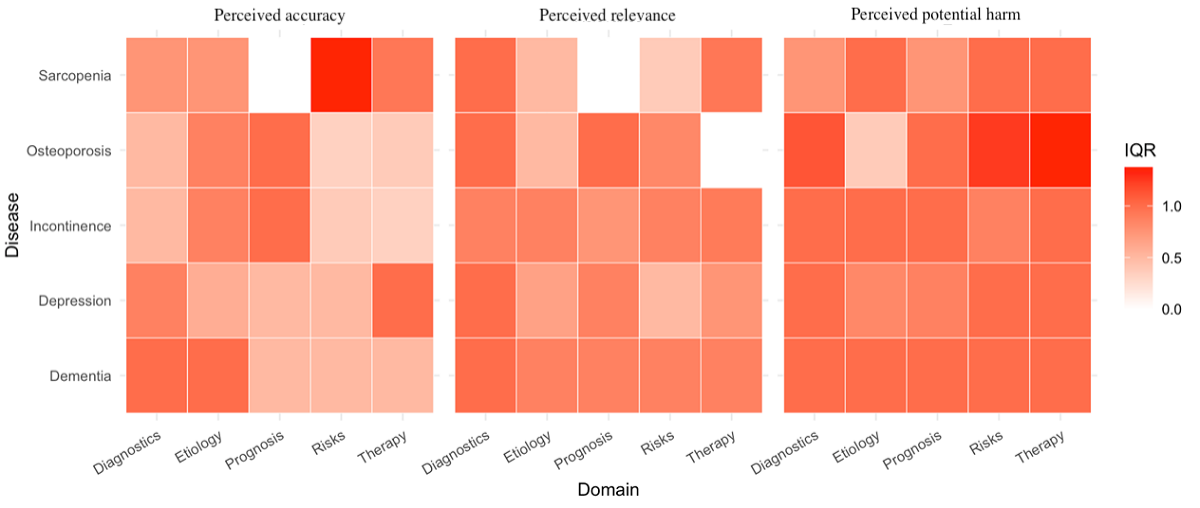
Heatmap depicting the dispersion of expert ratings based on the IQR of statement-level Likert ratings aggregated across raters. IQR values are shown across disease domains (sarcopenia, osteoporosis, urinary incontinence, depression, and dementia) and content domains (diagnostics, etiology, prognosis, risks of therapy, and therapy), stratified by rating dimension (perceived accuracy, perceived relevance, and perceived potential harm). Higher IQR values indicate greater dispersion in expert ratings, whereas lower values reflect more consistent assessments across statements.

An IQR-based analysis was performed to describe the dispersion of expert ratings across statements beyond measures of central tendency. Overall, IQR values were generally low to moderate across most disease and content domains (range 0.00-1.38), indicating limited variability in expert assessments (Figure 3).

Higher IQR values were observed in selected content domains. In particular, therapeutic statements related to osteoporosis showed greater dispersion in perceived potential harm ratings, as did risk-related statements in sarcopenia and osteoporosis. Additionally, moderate variability was observed for several diagnostic, etiological, and prognostic statements in dementia.

These descriptive patterns indicate that variability in expert ratings differed across content domains and statement types rather than being uniformly associated with specific diseases. Domains involving therapeutic decisions and risk appraisal tended to show greater dispersion, which may reflect heterogeneity in expert judgments.

### Agreement About Relative Ranking of Statements (Kendall W)

The Kendall coefficient of concordance (W) was calculated to explore agreement in the relative ordering of statements within the disease–content domain–dimension strata containing at least 3 statements. Of the 75 possible strata, 12 met the minimum requirement of 3 statements and were included in the analysis, while 63 strata were excluded due to insufficient item counts.

Across the 12 eligible strata, W values ranged from 0.27 to 0.62 (median 0.53, IQR 0.46-0.58), indicating moderate concordance in the relative ranking of statements by experts (Table 3). Even in strata with lower W values, median ratings remained high (Tables 1 and 2).

**Table 3:** Kendall coefficient of concordance (W) for all eligible disease–content domain–dimension strata containing at least 3 statements^a^.

Rating dimension and disease (content domain)			Kendall W (n statements)
**Perceived accuracy**			
	Depression (etiology)	0.406^b^	
	Osteoporosis (risks of therapy)	0.271^b^	
	Sarcopenia (therapy)	0.525^c^	
	Urinary incontinence (therapy)	0.287^b^	
**Perceived potential harm**			
	Depression (etiology)	0.620^b^	
	Osteoporosis (risks of therapy)	0.568^b^	
	Sarcopenia (therapy)	0.522^c^	
	Urinary incontinence (therapy)	0.623^b^	
**Perceived relevance**			
	Depression (etiology)	0.598^b^	
	Osteoporosis (risks of therapy)	0.549^b^	
	Sarcopenia (therapy)	0.479^c^	
	Urinary incontinence (therapy)	0.542^b^	

^a^Of the 75 possible strata, 12 met the minimum requirement for analysis (≥3 statements per stratum), while 63 strata were excluded due to insufficient item counts. W reflects agreement in the relative ordering of statements within each stratum and should be interpreted as exploratory, given the limited number of statements per group and the absence of a forced-ranking design.

^b^n=3.

^c^n=4.

Given the limited number of statements per stratum and the absence of a forced-ranking design, these findings should be interpreted as exploratory and descriptive rather than as definitive evidence of consensus or disagreement in clinical prioritization.

### Readability Analysis

Readability analysis indicated that LLM-generated responses were written at a median Flesch-Kincaid Grade Level of 8.3 (IQR 7.4-9.6), corresponding to grades 8 to 9 in the US educational system. The median Flesch Reading Ease score was 60.8 (IQR 50.1-66.9), indicating generally accessible language with moderate variability across responses.

## Discussion

### Principal Findings

In this exploratory expert rating study, geriatricians perceived LLM-generated patient information across 5 common geriatric conditions as largely accurate and relevant, with low potential harm when applied in typical informational contexts. Importantly, no statements combined low perceived accuracy with high perceived potential harm, suggesting that clearly misleading and dangerous content was not identified in the evaluated sample. Readability metrics suggested that the linguistic complexity of the responses was generally accessible for readers with secondary school literacy levels, although potentially challenging for individuals with limited health literacy. Nevertheless, variability in readability underscores the importance of considering health literacy and individual patient needs when deploying LLM-generated information in geriatric contexts [14].

The absence of statistically significant post hoc differences across diseases or content domains does not imply equivalence but reflects limited statistical power under multiple-testing correction. These results align with research in various medical disciplines that has similarly investigated perceived accuracy, relevance, and safety of AI-generated content [15,16]. Contrary to the extant literature on AI-generated patient education, which is predominantly single-condition and often limited to global quality or readability scores in areas such as sarcopenia [17], osteoporosis [18], urinary incontinence [19], depression [20], and dementia [21], our study provides a unified, cross-disease evaluation within a geriatric framework.

### Absolute Ratings Showed Limited Dispersion

Beyond comparisons of central tendency, the analysis of IQRs provided a complementary perspective on the variability of expert ratings. Overall, dispersion of ratings was limited across most disease and content domains, indicating broadly consistent expert assessments. However, greater variability was observed in selected content domains, particularly for therapeutic statements and statements addressing risks of therapy in sarcopenia and osteoporosis, as well as for several diagnostic, etiological, and prognostic statements in dementia.

Importantly, higher IQR values should not be interpreted as disagreement or lack of consensus but rather as reflecting heterogeneity in expert judgment. This pattern is clinically plausible, as therapeutic decision-making and risk appraisal often allow for a wider range of acceptable clinical perspectives, especially in complex geriatric contexts [22]. In particular, therapeutic and risk-related statements related to osteoporosis demonstrated higher variability as clinical guidelines and practice patterns (eg, osteoporosis medication thresholds [23]) vary, and clinical decision-making requires nuanced risk-benefit considerations [24-26]. Accordingly, the observed variability appeared more pronounced across content domains than across diseases.

### Moderate Concordance in the Relative Ranking of Statements

Kendall’s coefficient of concordance was used to explore agreement in the relative ordering of statements within disease–content domain–dimension strata containing at least 3 statements. Of the 75 possible strata, only 12 fulfilled the minimum requirement for analysis, highlighting the limited item counts within many domain combinations. Across eligible strata, W values ranged from 0.27 to 0.62 (median 0.53, IQR 0.46-0.58), indicating moderate concordance in the relative ranking of statements [27].

These findings should be interpreted cautiously. First, the small number of statements per stratum (typically 3-4) limits the stability of concordance estimates. Second, ratings were based on Likert-scale assessments rather than a forced-ranking task; therefore, Kendall W captures consistency in relative ordering derived from ordinal ratings rather than explicit prioritization decisions. Importantly, even in strata with lower concordance, median ratings for perceived accuracy and perceived relevance remained high, and perceived potential harm remained low. Thus, variability in ranking does not necessarily imply substantive disagreement regarding the overall quality or safety of the generated responses [28].

### Interpretation of Variability and Agreement

A central methodological consideration of this study is the distinction between variability in absolute ratings and agreement in relative ordering. Narrow IQRs indicate that experts tended to provide similar absolute judgments, whereas the Kendall coefficient of concordance reflects whether experts agreed on which statements were perceived as more or less acceptable relative to others [29]. These measures capture different dimensions of expert judgment and should not be interpreted interchangeably.

Observed variation in Kendall W across domains suggests heterogeneity in expert emphasis rather than disagreement regarding content validity. In domains such as therapy and risk-related information, clinicians may legitimately differ in how strongly they weight caution, nuance, or contextualization, even when overall perceived accuracy remains high. Given that relevance was assessed using Likert scales rather than a forced-ranking methodology, these findings should be interpreted as differences in perceived emphasis rather than as direct evidence of clinical prioritization [30].

### Methodological Considerations

This study relies on expert perception rather than objective verification against clinical guidelines or reference standards. Accordingly, the construct assessed here is *perceived* accuracy rather than *factual* correctness. This distinction is critical, as fluent and coherent LLM-generated text may receive favorable accuracy ratings despite containing subtle inaccuracies. Evidence from cognitive psychology indicates that processing fluency enhances perceived truthfulness, a phenomenon closely related to the illusory truth effect and fluency-based heuristics [31,32]. While expert ratings provide valuable insight into clinician judgment, they cannot substitute for systematic guideline-based validation [33].

Agreement analyses using Kendall W were conducted exploratorily and restricted to strata with a minimum number of statements. Nevertheless, the limited number of items within several strata constrains the stability of concordance estimates and warrants cautious interpretation. These analyses are best understood as descriptive signals of agreement patterns rather than definitive measures of consensus.

### Limitations

Several limitations should be considered. First, the expert panel was relatively small and geographically homogeneous, consisting exclusively of geriatricians from Germany, which may limit generalizability to other health care systems and guideline contexts. Second, the selection of statements was based on commonly encountered clinical questions, which likely biases the evaluation toward well-established and comparatively “safe” topics. Performance in rare, complex, or atypical scenarios, where LLM hallucinations may be more consequential, was not assessed [10]. As question selection was guided by clinical relevance and frequency rather than equal representation across predefined domains, the resulting distribution of items was inherently unbalanced. This led to the exclusion of multiple strata from the Kendall coefficient of concordance analysis due to insufficient item counts.

Third, although relevance was rated favorably by clinicians, readability metrics indicated that the linguistic complexity of responses may exceed the health literacy level of geriatric patients. This gap highlights the importance of integrating readability optimization and patient-centered evaluation in future assessments of LLM-generated medical information [34]. Additionally, many generated responses were relatively short. As readability formulas such as the Flesch Reading Ease and Flesch-Kincaid Grade Level are sensitive to text length, their application to short text passages (eg, <100 words) may yield unstable or less reliable estimates.

Fourth, the study evaluated single-turn, zero-shot LLM responses and did not capture interactive dialogue, follow-up clarification, or longitudinal consistency, all of which are central to real-world patient information seeking. In addition, the limited number of responses within several disease-content domain strata restricted agreement analyses and reduced the stability of concordance estimates. Finally, relevance was assessed mostly from a clinician’s perspective; readability was assessed, but patient comprehension or usability were not evaluated and warrant future investigation [35].

### Implications and Future Directions

From an AI evaluation perspective, these findings suggest that expert-based perception studies can serve as an initial filter for identifying potentially problematic content in patient-oriented LLM outputs. However, such assessments should be complemented by objective validation approaches, including guideline-based accuracy checks and studies incorporating patient-centered outcomes. Future work should also examine more complex clinical scenarios and interactive use cases to better reflect real-world deployment.

### Conclusions

In this exploratory expert rating study, LLM-generated patient information for common geriatric conditions was perceived as largely accurate and relevant, with low perceived potential harm in typical clinical scenarios. Variability in expert judgments primarily reflected differences in emphasis rather than overt disagreement on content validity. Given the perception-based nature of the assessment, limited item counts for agreement analyses, and the absence of objective reference standards, these findings should be interpreted cautiously. Future evaluations should combine expert ratings with guideline-based validation and patient-centered outcome measures to more comprehensively assess the safety and suitability of LLM-generated information for geriatric patient education.

## Appendix

Multimedia Appendix 1 Supplementary material including all geriatric patient questions with corresponding ChatGPT-generated responses and characteristics of expert reviewers.

## Abbreviations

AI: artificial intelligence
LLM: large language model

## Data Availability

The datasets generated and analyzed during this study are available from the corresponding author on reasonable request.
