# Supplementary material for: Expert Evaluation of the Perceived Accuracy, Relevance, and Safety of Large Language Model–Generated Patient Information in Geriatrics: Cross-Condition Study
Source: JMIR AI. 2026 May 4;5:e91369. doi: 10.2196/91369 (PMC13184597; doi:10.2196/91369)
Supplement: Multimedia Appendix 1 [file ai_v5i1e91369_app1.docx]

**Multimedia Appendix 1:**

Supplementary material including all geriatric patient questions with corresponding ChatGPT-generated responses and characteristics of expert reviewers.

**Supplementary Tables**

|  | **Question** | **Answer** |
| --- | --- | --- |
| 1 | Is osteoporosis simply a normal part of aging? | No, osteoporosis is not just a normal part of aging. Bones do get thinner as we get older, but osteoporosis means they become much weaker than usual and break more easily. Many older adults keep their bones strong with healthy habits and treatment if needed. |
| 2 | Can medications cause osteoporosis? | Yes, some medicines can weaken bones if you take them for a long time. Examples include certain steroids (like prednisone), some cancer treatments, or medicines for seizures. Not everyone who takes these will get osteoporosis, but your doctor can watch your bone health and help protect you if you need those medicines. |
| 3 | Why do I lose muscle as I get older? | As we get older, our bodies naturally make less muscle and our hormones change. We also tend to move less, which makes the muscles shrink and weaken faster. Illness and poor nutrition can add to this. |
| 4 | Can medications cause muscle loss? | Yes, some medicines can cause muscle loss or weakness, especially if you take them for a long time. Examples include certain steroids, some cancer drugs, or medicines for the heart. |
| 5 | Isn't dementia just a normal part of getting older? | No, dementia is not a normal part of aging. As we get older, it’s normal to forget things occasionally. Dementia, however, causes stronger and progressive problems with memory, thinking, and daily life. That’s why it’s important to check carefully if changes go beyond normal aging. |
| 6 | Can medications cause dementia? | Some medicines can cause memory problems or confusion that look like dementia, especially in older people. Often, these symptoms improve when the medication is changed or stopped. True dementia is different—it doesn’t go away. That’s why we always review your medicines carefully if memory problems appear. |
| 7 | Isn’t it normal to feel sad or tired at my age? | It’s normal to feel sad or tired sometimes, especially with age or illness. But lasting sadness, loss of joy, or strong fatigue are not just “normal aging.” These may be signs of depression, which is treatable. It’s important to talk about such symptoms so we can help. |
| 8 | What if this is just loneliness, grief or boredom? | Loneliness, grief, or boredom can cause sadness and low energy, but depression is stronger and lasts longer. Even if these feelings start from life events, depression can develop. The good news: both depression and the effects of loneliness or grief can be treated and supported—so it’s always worth talking about. |
| 9 | Can medications cause depression? | Yes, some medicines can cause low mood or depression-like symptoms, especially in older adults. Often, these improve when the drug is changed or stopped. If you feel unusually down after starting a new medication, tell your doctor so we can check and adjust your treatment safely. |
| 10 | Isn't incontinence just a normal part of getting older? | No, incontinence is not just a normal part of aging. While bladder control problems become more common with age, they often have specific causes—like prostate changes, weak pelvic muscles, or medications. Many cases can be improved or treated, so it’s important to talk about it and not just accept it. |
| 11 | Can medications cause incontinence? | Yes, some medicines can affect the bladder or kidneys and lead to incontinence, such as water pills, sedatives, or drugs for blood pressure. These effects may improve if the medication is adjusted. Always tell your doctor about bladder changes so we can check your medicines and find solutions. |
| 12 | How do you find out if I have osteoporosis? | We usually check for osteoporosis with a simple, painless scan called a bone density test (DEXA scan). It measures how strong your bones are. Your doctor may also ask about past falls, broken bones, or family history. Sometimes, blood tests are done to rule out other causes of weak bones. |
| 13 | I had a bone fracture – does that automatically mean I have osteoporosis? | Not always. Breaking a bone doesn’t automatically mean you have osteoporosis. Sometimes fractures happen from a bad fall or accident. But if the break happened from a small bump, slip, or even just twisting the wrong way, that can be a sign your bones are weaker than normal. In that case, your doctor would check you with a bone density test to see if it’s osteoporosis. |
| 14 | How do you find out if I have sarcopenia? | We find out if you have sarcopenia by checking your muscle strength and function. Your doctor may test how strong your grip is, how fast you can walk, or how easily you can get up from a chair. Sometimes, a scan is used to see how much muscle you have. |
| 15 | How do you find out if I have dementia? | We find out if you have dementia by talking with you and someone close to you, doing short memory and thinking tests, checking for other causes with blood tests or scans, and sometimes referring you to a specialist. The goal is to see what’s behind your symptoms and if treatment is possible. |
| 16 | Can dementia also be detected through a blood test? | No, dementia itself cannot be diagnosed with a blood test. Blood tests help rule out other causes of memory problems, like vitamin deficiencies or thyroid issues. Dementia is mainly detected through memory tests, medical history, and sometimes brain scans. |
| 17 | How do you find out if I have depression? | To find out if you have depression, I talk with you about mood, sleep, appetite, and daily energy. I may ask short questions or use a questionnaire. Blood tests can rule out other causes like thyroid problems. This helps us see if it’s depression and how best to treat it. |
| 18 | Will I have to go to a specialist or a hospital for the diagnosis of depression? | Usually, depression can be diagnosed by your family doctor through conversation and simple questions. A specialist or hospital is only needed if symptoms are severe, unclear, or other health problems are involved. Most people don’t need hospital care to find out if they have depression. |
| 19 | Do I need a bladder scan, urine test, or something more invasive for the diagnosis of incontinence? | To check incontinence, we usually start with a talk, a bladder diary, and a urine test. Sometimes a simple bladder scan is added. More invasive tests are only needed if the cause is unclear or symptoms are severe. Most people don’t need complicated procedures at first. |
| 20 | Will I have to go to a specialist or a hospital for the diagnosis of incontinence? | Most incontinence can be assessed by your family doctor with questions, a urine test, and sometimes a bladder scan. A specialist or hospital is only needed if the cause is unclear, symptoms are severe, or first treatments don’t help. Often, diagnosis and care start right at your doctor’s office. |
| 21 | Is the therapy for osteoporosis still worthwhile if I am very old? | Yes, it is still worthwhile. Even at your age, treatment can make your bones stronger and lower the chance of a serious fracture, like a hip break. A bad fracture could mean pain, hospital stays, or losing independence. |
| 22 | Is the therapy for sarcopenia still worthwhile if I am very old? | Yes, therapy is still worthwhile even if you are very old. Exercise and good nutrition can help you stay stronger, lower your risk of falls, and keep your independence longer. |
| 23 | Will I lose my independence if I have dementia? | Dementia affects independence slowly. In the beginning, many people still manage daily life with some support. Over time, more help is often needed. The goal of treatment and support is to keep you independent and safe as long as possible, while planning ahead for future needs. |
| 24 | How fast will dementia get worse? | Dementia usually worsens slowly over years, but the speed is different for each person. Some notice changes gradually, others more quickly. Treatment, healthy lifestyle, and good support can sometimes slow the decline and help maintain quality of life longer. |
| 25 | Can I stop antidepressants if I feel better? | You shouldn’t stop antidepressants on your own, even if you feel better. Stopping too quickly can cause symptoms to return or withdrawal effects. Always talk to your doctor first—we can decide together when and how to reduce the medication safely. |
| 26 | Will people think I’m mentally ill when I am diagnosed with depression? | Depression is a common medical condition, not a weakness or shame. Many people your age experience it, and treatment helps. Having depression does not mean you’re “crazy” or less valued—it means you’re facing an illness that deserves care, just like diabetes or heart disease. |
| 27 | Will incontinence affect my ability to live independently? | Incontinence doesn’t automatically mean losing independence. With the right treatment, many people manage symptoms well. Planning ahead with toilet access, using protective products, and getting medical support can keep you active and independent. The key is to talk openly so we can find solutions together. |
| 28 | Do medications for osteoporosis have side effects? | Yes, like most medicines, osteoporosis drugs can have side effects. Common ones may include stomach upset, heartburn, or muscle aches. Serious side effects are rare. Your doctor will choose the medicine that’s safest for you and keep an eye on how you’re doing. |
| 29 | Bisphosphonates: Why do I have to sit or stand up after taking it? | You need to stay sitting or standing after taking bisphosphonates so the pill goes straight down into your stomach. If you lie down, it could get stuck in your food pipe and cause burning or irritation. |
| 30 | Bisphosphonates: I heard something about jaw problems—is that true? | Yes, but it’s very rare. A small number of people can get jawbone problems, usually if they’ve had dental surgery. Your doctor will check your teeth and lower your risk. |
| 31 | Is exercise safe for someone my age or with my health problems? | Yes, exercise is safe at your age, even with health problems—as long as it’s tailored to you. A doctor or therapist can guide you to start slowly and choose safe movements. Exercise can actually improve your health, balance, and independence. |
| 32 | Do protein supplements cause stomach problems? | Sometimes protein supplements can cause stomach problems like bloating, gas, or diarrhea, especially if taken in large amounts. Many people tolerate them well, and changing the type or taking smaller portions can help. |
| 33 | Do medications for dementia have side effects? | Yes, dementia medicines can have side effects, such as nausea, diarrhea, dizziness, or sleep problems. Not everyone gets them, and they’re often mild or improve over time. We always weigh the benefits against the risks and adjust treatment if side effects become troublesome. |
| 34 | Can dementia medication cause problems with my bladder or bowel habits? | Yes, some dementia medicines can cause bladder or bowel problems, like needing to urinate more often, accidents, constipation, or diarrhea. Not everyone experiences this, and sometimes adjusting the medication helps. If such problems occur, it’s important to tell your doctor so we can find the best solution. |
| 35 | Do medications for depression have side effects? | Yes, depression medicines can cause side effects, like nausea, dizziness, dry mouth, or sleep changes. Many are mild and improve with time or dose adjustment. Not everyone gets them, and we always balance benefits and risks to find the treatment that suits you best. |
| 36 | Can antidepressants cause weight gain or loss? | Yes, some antidepressants can lead to weight gain, while others may cause weight loss. Not everyone is affected, and the changes are often small. If weight shifts become a concern, we can adjust the medication or support you with diet and activity to help manage it. |
| 37 | Do medications for incontinence have side effects? | Yes, medicines for incontinence can cause side effects like dry mouth, constipation, blurred vision, or dizziness. Not everyone gets them, and sometimes they fade with time. If side effects are bothersome, we can adjust the dose or switch to another treatment option. |
| 38 | Are there surgical treatments – and what are the risks? | Yes, there are surgical options for certain types of incontinence, such as slings or procedures to support the bladder. Surgery is usually only considered if other treatments don’t help. Risks include infection, pain, or not fully solving the problem. We carefully weigh benefits and risks before recommending it. |
| 39 | Why should I treat osteoporosis if I don't have any pain? | Osteoporosis usually doesn’t cause pain until a bone breaks. Treating it early helps prevent fractures in the hip, spine, or wrist, which can lead to pain, loss of independence, or serious health problems. |
| 40 | How long does it take for the treatment for osteoporosis to work? | Osteoporosis treatment starts helping your bones right away, but it usually takes several months to a year before we can see stronger bones on a scan. The good news is that the medicine lowers your risk of fractures as you keep taking it. |
| 41 | What about exercise: does physical or occupational therapy really help in sarcopenia? | Yes, exercise and therapy really do help. Special exercises, like strength and balance training, can slow down muscle loss, make you stronger, and lower your risk of falls. Therapists can also show you safe ways to stay active in daily life. |
| 42 | How long does it take for the treatment for sarcopenia to work? | Treatment for sarcopenia takes time. With regular exercise and good nutrition, you may start to feel stronger in a few weeks, but bigger improvements often take 2–3 months or more. |
| 43 | Are there any medications for sarcopenia? | Right now, there are no specific medicines approved just for sarcopenia. Doctors focus on exercise, good nutrition, and enough protein to protect your muscles. Sometimes other treatments, like vitamin D or hormone therapy, may be used if needed. |
| 44 | I already stay active in my daily life – isn't that enough for sarcopenia? | Staying active in daily life is very good, but it may not be enough to keep muscles strong. For sarcopenia, you need specific exercises like lifting light weights, using resistance bands, or doing balance training. These work your muscles more than just walking or housework. |
| 45 | Does memory training even help at all for dementia? | Memory training cannot stop dementia, but it can help keep your mind active, support daily functioning, and sometimes slow down how quickly problems get worse. Staying mentally, physically, and socially active is beneficial and can improve quality of life. |
| 46 | How long does it take for the treatment for dementia to work? | Treatment for dementia doesn’t cure the disease, but certain medications or therapies can ease symptoms or slow worsening. Effects vary: some people notice improvements within weeks or months, while for others the benefit is modest. Supportive measures—like routines, exercise, and social activity—help right away in daily life. |
| 47 | How long does it take for the treatment for depression to work? | Depression treatment takes time. Medications often need 2–6 weeks before you feel better. Talking therapies and lifestyle changes can also help but work gradually. Some people notice improvements sooner, others later. We monitor closely and adjust treatment to find what works best for you. |
| 48 | How long does it take for the treatment for incontinence to work? | Treatment time depends on the cause. Bladder training or pelvic exercises may help within weeks, while medications can take a few days to weeks. Sometimes finding the best approach takes longer. With patience and the right plan, many people notice clear improvement over time. |
| 49 | Can incontinence be treated at all? | Yes, incontinence can often be treated or improved. Options include bladder training, pelvic floor exercises, lifestyle changes, medications, or minor procedures. The best approach depends on the cause. While it may not always be cured, many people gain much better control and quality of life with treatment. |
| 50 | Will I need therapy for incontinence forever, or is this just temporary? | It depends on the cause. Some people improve with short-term therapy, like bladder training or medication, and may not need long-term treatment. Others benefit from ongoing exercises or support to keep symptoms under control. We tailor the plan to your needs and adjust it over time. |

***Supplementary Table S1*** *All 50 questions from geriatric patients and responses generated by ChatGPT (version 5.1). Frequently asked questions encountered in routine geriatric practice were selected by panel members using a predetermined point-based system that reflected their perceived frequency in routine clinical practice. Responses were generated by ChatGPT using a standardized input procedure.*

| **Characteristics of Reviewers** | | | | |
| --- | --- | --- | --- | --- |
|  | | | N=10 | |
| Years as a geriatrician | <5 | 5-10 | 10-15 | >15 |
| Numbers | 2 | 2 | 3 | 3 |
| Geriatric patients treated **in hospital** annually for | <20 | 20-50 | 50-100 | >100 |
| Osteoporosis | 1 | 0 | 1 | 8 |
| Sarcopenia | 0 | 0 | 1 | 9 |
| Dementia | 0 | 1 | 1 | 8 |
| Depression | 0 | 2 | 0 | 8 |
| Incontinence | 0 | 1 | 1 | 8 |
| Geriatric patients treated **personally** annually for |  |  |  |  |
| Osteoporosis | 1 | 2 | 3 | 4 |
| Sarcopenia | 0 | 0 | 6 | 4 |
| Dementia | 0 | 3 | 1 | 6 |
| Depression | 0 | 4 | 1 | 5 |
| Incontinence | 2 | 1 | 3 | 4 |

***Supplementary Table S2*** *Characteristics of reviewers. Years as a geriatrician were recorded using ordinal categories. The median category was 10–15 years. The number of geriatric patients treated in hospital and the number of patients personally treated for osteoporosis, sarcopenia, dementia, depression, and urinary incontinence were also collected using ordinal response categories and are presented as numbers.*
